# Critical factors influencing physicians’ intention to use computerized clinical practice guidelines: an integrative model of activity theory and the technology acceptance model

**DOI:** 10.1186/s12911-016-0241-3

**Published:** 2016-01-16

**Authors:** Ju-Ling Hsiao, Rai-Fu Chen

**Affiliations:** 1 Department of Hospital and Health Care Administration, Chia-Nan University of Pharmacy and Science, Tainan City, Taiwan R.O.C.; 2 Department of Information Management, Chia-Nan University of Pharmacy and Science, No.60, Sec. 1, Erren Rd., Rende Dist., Tainan City, 71710 Taiwan R.O.C.

**Keywords:** Activity theory, Attitude to computers, Clinical practices guideline systems, Evidence-based medicine, Technology acceptance model

## Abstract

**Background:**

With the widespread use of information communication technologies, computerized clinical practice guidelines are developed and considered as effective decision supporting tools in assisting the processes of clinical activities. However, the development of computerized clinical practice guidelines in Taiwan is still at the early stage and acceptance level among major users (physicians) of computerized clinical practice guidelines is not satisfactory. This study aims to investigate critical factors influencing physicians’ intention to computerized clinical practice guideline use through an integrative model of activity theory and the technology acceptance model.

**Methods:**

The survey methodology was employed to collect data from physicians of the investigated hospitals that have implemented computerized clinical practice guidelines. A total of 505 questionnaires were sent out, with 238 completed copies returned, indicating a valid response rate of 47.1 %. The collected data was then analyzed by structural equation modeling technique.

**Results:**

The results showed that attitudes toward using computerized clinical practice guidelines (*γ* = 0.451, *p* < 0.001), organizational support (*γ* = 0.285, *p* < 0.001), perceived usefulness of computerized clinical practice guidelines (*γ* = 0.219, *p* < 0.05), and social influence (*γ* = 0.213, *p* < 0.05) were critical factors influencing physicians’ intention to use computerized clinical practice guidelines, and these factors can explain 68.6 % of the variance in intention to use computerized clinical practice guidelines.

**Conclusions:**

This study confirmed that some subject (human) factors, environment (organization) factors, tool (technology) factors mentioned in the activity theory should be carefully considered when introducing computerized clinical practice guidelines. Managers should pay much attention on those identified factors and provide adequate resources and incentives to help the promotion and use of computerized clinical practice guidelines. Through the appropriate use of computerized clinical practice guidelines, the clinical benefits, particularly in improving quality of care and facilitating the clinical processes, will be realized.

## Background

### Current status of clinical practice guidelines in Taiwan

Clinical practice guidelines (CPGs) are “systematically developed statements to assist practitioner and patient decisions about appropriate health care for specific clinical circumstances” [1]. Many studies have considered CPGs to be standardized clinical models developed on the basis of evidence-based medicine (EBM) for improving the quality of care and reducing variations in clinical practice at affordable costs [1–4]. Therefore, the effective use of CPGs can help health care professionals, particularly physicians, make appropriate health care decisions, thus improving the quality of care. In Taiwan, the concept of CPGs was introduced by the National Health Insurance Administration in 2003 to address the dilemma between medical quality and cost. Until 2008, there were 17 clinical guidelines developed under the assistance of various medical associations in Taiwan. However, each of these clinical guidelines was developed to provide guidelines for a single disease rather than complex problems of comorbidity and multimorbidity [5–8].

Even if a CPG is well developed, it might not be implemented appropriately [9–13]. Handler and Lackland [11], Mourad et al. [14], and Schnoor et al. [15] have indicated that implementing multiple strategies rather than a single strategy is more effective for the widespread use of CPGs. Feasible implementation strategies for promoting CPGs include education and training, leaflets or manuals, proactive reminder systems, and monitor and feedback of results. Numerous medical institutions suggest that physicians use paper-based clinical guidelines; however, those that are paper based are inconvenient and time consuming to read, and fail to help physicians making clinical decisions in a timely manner. To address the problems mentioned, computerized CPGs were developed, implemented, and integrated into existing hospital information systems (HIS) or electronic medical record (EMR) systems by selected Taiwanese hospitals [6]. Although it was confirmed that computerized CPG use significantly improves the process of care [16], the development of computerized CPGs in Taiwan is still at the early stage and acceptance level among major users (physicians) of computerized CPGs is not satisfactory. Physicians are the key providers of health care services and the principal users of computerized CPGs; thus, physicians’ intention to use computerized CPGs is critical for the success of computerized CPGs. To explore the critical factors influencing physicians’ intention to use computerized CPGs and to provide an understanding of the underlying hindrances to computerized CPG use, this study conducted a survey in three hospitals that have implemented computerized CPGs.

### Studies on CPGs and computerized CPGs

Lyng [17] summarized four types of guidance that CPGs provide for health care professionals: decision support, process support, documentation support, and task support. Lyng [17], Grimshaw et al. [18], and Johnson and Turley [19] have mentioned that only a few CPGs are actively used, despite being proposed, recommended, and applied in clinical practice. One possible explanation is that CPG use may result in variations in the clinical behaviors of physicians, who can become resistant to their continued use [20, 21]. Larson [22] indicated that more attention should be paid to applying CPGs in clinical practice. Many studies have focused on promoting CPG use in clinical practice by emphasizing topics related to CPG development [23–25], the establishment of interchange standards for computerized CPGs [26, 27], and computerized CPG design and implementation [28–39]. Some of these studies have focused on solving comorbidity and multimorbidity problems [28, 29, 34, 39] by incorporating knowledge-based techniques such as semantic web or constraint logic. Although feasible suggestions have been proposed for solving comorbidity and multimorbidity problems, obstacles still hinder computerized CPG development. Furthermore, Isern and Moreno [37] indicated that computerized CPGs could benefit clinicians and patients, but the systems were not yet fully integrated and implemented into existing careflow management systems and thus not used in daily clinical practice. Lyng [17] argued that most computerized CPGs are technology driven [37], and only a few studies have focused on a user-center approach in the design of CPGs [30, 38].

In addition, some studies have investigated factors influencing the implementation of CPGs for health care professionals through a systematic metareview approach, to address managerial concerns about CPG use [40–42]. Francke et al. [40] categorized the influencing factors of CPG use as follows: 1) characteristics of the guidelines (e.g., being easy to understand and implement, without requiring specific resources), 2) characteristics of the implementation strategies (e.g., effective strategies often have multiple components rather than a single strategy), 3) characteristics of professionals (e.g., awareness of the existence of a guideline and familiarity with its content), 4) characteristics of patients (e.g., comorbidity reduces the chance that guidelines are followed), and 5) characteristics of the environment (e.g., impediments such as a lack of support from peers or superiors as well as insufficient staff and time). Kortteisto et al. [41] indicated that attitudes toward the behavior, subjective norm, and perceived behavioral control were crucial factors associated with professionals’ intention to use CPGs. Flottorp et al. [42] identified seven domains influencing health care professional practices: guideline factors; individual health professional factors; patient factors; professional interactions; incentives and resources; capacity for organizational change; and social, political, and legal factors. As mentioned, these factors have provided insights into further research on computerized CPGs and the careful evaluation of the effects of their use.

### Theoretical foundations

#### Activity theory

Activity theory was first proposed by Vygotsky for understanding the mental capabilities of an individual through in-depth analysis of the cultural and technical aspects of human actions [43]. Kuutti [44] argued that activity theory is a cross-disciplinary framework for studying different forms of human practice, factoring in the processes of context as a developmental process, at both the individual and social levels simultaneously, including the use of artifacts. According to Leontiev [45], the core of activity theory is activity, which is composed of a subject, object, actions, and operations. Nardi [46] argued that “activities are realized as individual and cooperative actions, and chains and networks of such actions, related to each other by the same overall object and motive.” In addition, activity theory focuses on social factors and the interaction between subjects and their environments and explains why the principle of tool mediation plays a central role in the activity approach. Tools are created and transformed during the development of an activity and vary depending on the culture. Therefore, tools constitute a means of accumulating and transmitting social knowledge.

Recently, activity theory has been extended to the fields of the human-machine interface [47], learning [48, 49], e-commerce [50], intensive care patient discharge [51], and computerized diabetes disease management [52]. Most of the cited studies have applied activity theory as a practical and descriptive framework for facilitating the understanding of the interrelationships among activity systems through descriptive and qualitative research methodologies. Sun et al. [53] suggested that user behavior is closely related to the context in which the user is placed (e.g., social environment). Activity theory regards the information system as a manmade artifact or tool and is applied to analyze the interactive relationships between human behavior and social associations [46]. In the context of the health care industry (environment), a computerized CPG (tool) is viewed as a type of manmade information tool or a clinical decision-making system used by physicians (subjects) to obtain real-time information for completing clinical activities and decisions, to improve the quality of clinical decisions, and to ensure patient safety (objects). In addition, the use of computerized CPGs involves learning adaptive processes that entail numerous activities. Therefore, activity theory was adopted as the research framework of this study to provide insights into the potential reasons for and factors influencing computerized CPG use according to the dimensions of the environment, tool, and subject; these factors are critical for realizing the benefits of computerized CPGs (object). The identified factors found in prior CPG studies can be divided into three characteristics mentioned in activity theory for further validation in the context of computerized CPGs. Yusof et al. [54] proposed a similar research framework (HOT-fit) of activity theory and argued that successful healthcare information technologies (HIT) implementation depends on a good fit among human, organizational, and technical elements. Both activity theory and HOT-fit emphasize the interaction among the subject (human), environment (organization), and tool (technology).

#### Technology acceptance model

The technology acceptance model (TAM), developed by Davis [55], is one of the most used theoretical models for predicting and explaining whether users will accept new information technology (IT)/information systems (IS). The model has argued that people’s attitudes toward behaviors and subjective criteria determine their behavioral intentions toward technology applications, which consequently affect their own behavior [55]. Perceived usefulness and perceived ease of use are two major factors influencing user IT acceptance (intention/attitude/use), and the two factors are influenced by many external variables. Although numerous TAM-related studies have investigated various groups and application systems, Wu et al. [56] indicated that the TAM focuses more on technology aspects without considering the effects on human and organizational factors in the adoption process. Yarbrough and Smith [57] indicated that TAM constructs generally hold in a physician-specific context, but the perceived ease of use component of the model does not prove to be consistently related to either attitudes or perceived usefulness. Yarbrough and Smith [57] argued that one limitation of the TAM is its inability to consider the influence of external variables and barriers to technology acceptance. They further suggested customizing the inclusion of variables (personal characteristics, organizational characteristics, and IS characteristics) to enhance the model’s accuracy.

Recently, some studies have proposed an extended TAM, incorporating considerations of human, organizational, and technology factors based on the TAM, for investigating critical factors influencing both user (i.e., health care professional) acceptance of HIS [54] and pain management decision support systems [58]. The two cited studies have significantly improved (84.4 and 64 %, respectively) on the variance of user acceptance. By contrast, some studies have investigated physicians’ acceptance of health care systems by using the unified theory of acceptance and use of technology (UTAUT) model or modified UTAUT model [59–61]. The UTAUT model identifies four key constructs (performance expectancy, effort expectancy, social influence, and facilitating conditions) as direct determinants of user intention and behavior, and four moderators (gender, age, experience, and voluntariness of user) [62]. However, the variance of physicians’ user acceptance (intention) of the TAM, UTAUT, or revised UTUAT model was approximately 20–47 %. For example, Venkatesh et al. [61] indicated that the original UTUAT predicted only approximately 20 % of the variance in intention, and the modified UTAUT predicted 44 %. This indicated that some predictors of physicians’ intention remain unknown and further investigation is necessary. Our study explored the critical factors influencing physicians’ intention to use computerized CPGs on the basis of the extended TAM concept rather than the original UTAUT.

## Methods

### Research framework

**Fig. 1:**
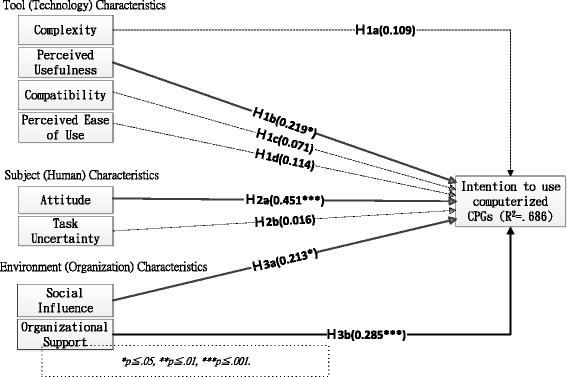
Results of model validity regarding factors affecting physicians’ intention to use computerized CPGs

We propose an integrated research model for exploring and understanding critical factors influencing physicians’ intention to use computerized CPGs by incorporating activity theory (three dimensions of factors) with TAM concepts (intention as dependent variable). The investigated variables of the model are derived from results obtained in previous TAM and CPG studies. The model is composed of three independent dimensions: tool (technology) characteristics (i.e., complexity, perceived usefulness, compatibility, and perceived ease of use), subject (human) characteristics (i.e., attitude and task uncertainty), and environment (organization) characteristics (i.e., social influence and organizational support). These dimensions with eight variables are considered potential critical factors (independent variable) influencing physicians’ intention to use computerized CPGs. Relevant studies on the model are summarized in Table 1, and the research model is depicted in Fig. 1.

**Table 1:** Relevant studies to the dimensions of the research model

Dimension (variable)	Francke et al. [40]	Flottorp et al. [42]	Kortteisto et al. [41]	UTAUT related studies [59–61]
Tool (Technology)				
Complexity	Easy to understand, can easily be tried out, and do not require specific resources	Guideline factors		
Perceived usefulness				Performance expectancy
Compatibility	Effective strategies often have multiple components than single strategy			
Perceived ease of use				Effort expectancy
Subject (Human)				
Attitude	Awareness of the existence of the guideline and familiarity with its content	Individual health professional factors	Attitude toward the behavior	
Task uncertainty		Individual health professional factors		
Environment (Organization)				
Social influence	A lack of support from peers or superiors	Professional interactions	Subjective norm	Social influence
Organizational support	Insufficient staff and time	Incentives and resources, capacity for organizational change, and social, political, and legal factors	Perceived behavior control	Facilitating conditions

The relationships between activity theory and the three dimensions of factors investigated in this study are summarized as follows: 1) The ultimate goals (objects) of computerized CPG use are to improve the quality of care and to provide timely decision support; however, these goals are achievable only when the computerized CPGs are implemented and used widely by their major users (physicians); 2) physicians’ intention to use computerized CPGs is a surrogate for evaluating the successful implementation of computerized CPGs (user behavior); 3) the three dimensions (tool, subject, and environment/organization) of activity theory are critical for achieving expected goals obtained from computerized CPG use; 4) a tool is a characteristic of computerized CPGs, and four factors (complexity, perceived usefulness, compatibility, and perceived ease of use) are identified; 5) subjects are characteristics of the major users (physicians) of computerized CPGs, and two factors (attitude and task uncertainty) are identified; and 6) the environment/organization is the context in which users (physicians) are placed, and two factors (social influence and organizational support) are identified.

The tool characteristics of the research model are defined as follows: (1) Complexity is the degree to which the content and presentation of computerized CPGs is difficult to understand and read [40, 63]; (2) perceived usefulness represents users’ subjective beliefs about the benefits of using HIT to achieve job goals in medical practice [64, 65]; (3) compatibility refers to the degree to which the system is consistent with the user’s value, demand, and experience [65, 66]; and (4) perceived ease of use represents the degree to which users believe that using HIT is free of effort [57, 65]. Some studies [40, 42] have indicated that computerized CPGs are difficult to understand and that using them requires certain resources; thus, computerized CPGs have a low likelihood of being adopted by users. Thus, the high complexity of computerized CPGs and the substantial effort and time spent by users in learning how to use them inhibit computerized CPG use. Perceived usefulness was found to be a critical factor influencing the user acceptance of systems among various health care professionals [58–61, 65, 67]. When physicians perceive a higher degree of system usefulness (i.e., real-time information and benefits in improving quality and physicians’ training and education), they have a more positive attitude and are willing to accept computerized CPGs.

Rogers [63] defined compatibility as the degree to which a newly developed system conforms to the organizational culture, individual experiences, and user requirements. Previous studies have indicated that compatibility is a critical factor affecting users’ willingness to adopt innovative technology [68, 69]. A newly developed system that highly conforms to the original organizational culture and user requirements is highly compatible and easy to accept. Hence, we assume that the compatibility of computerized CPGs with the original organizational system and physicians’ work experience, practice, and culture positively influences physicians’ willingness to use them. In addition, Venkatesh et al. [62] argued that IS is easy to use and can enhance performance. When physicians perceive that learning how to use CPGs is easy, they have a more positive attitude toward accepting computerized CPGs and a higher intention to use computerized CPGs. Therefore, computerized CPGs that have an easy-to-operate interface require little time and effort for learning, require no dedicated personnel for education and training, and have a high probability of being adopted [70]. Perceived ease of use was found to be a critical factor influencing the user acceptance of systems among various health care professionals [58, 65, 67].

The subject characteristics are defined as follows: (1) Attitude is an individual’s positive or negative perception of an object, an action, or a person [71], and (2) task uncertainty refers to the degree of uncertainty and unstructured conditions faced by physicians in clinical work [72, 73]. Saillour-Glenisson and Michel [74] have indicated that a lack of knowledge, familiarity, and recognition causes a negative physician attitude toward CPGs. They found that the degree of structure of the work environment is a crucial factor affecting the physicians’ CPGs use. Kortteisto et al. [41] found that health care professionals’ attitude toward a behavior was a critical factor associated with the intention to use CPGs. Therefore, we hypothesized that uncertainty and unstructured clinical work conditions positively influence physicians’ intention to use computerized CPGs.

The environment characteristics are defined as follows: (1) Social influence represents the degree to which an individual perceives that important others (i.e., colleagues or supervisors) believe that he or she should use a system [62, 75], and (2) organizational support refers to resources (time, money, and human) provided by health care institutions to physicians to alleviate stress during computerized CPG use [76]. Francke et al. [40] and Flottorp et al. [42] have argued that support from colleagues or supervisors is critical for facilitating CPG use. Moreover, Kortteisto et al. [41] determined that the subjective norm, a concept similar to social influence, is a crucial factor associated with health care professionals’ intention to use CPGs. Therefore, we hypothesized that a supervisor and colleague having a positive attitude toward computerized CPG use and actively providing a supporting environment positively influence physicians’ willingness to use computerized CPGs. Organizational resources include human resources, support provided by relevant facilities, and time for diagnosis and treatment. Francke et al. [40], Flottorp et al. [42], and Kortteisto et al. [41] have suggested that organizational resources are critical for CPG use. Hence, we hypothesized that medical institutions providing adequate human resources and sufficient time to reduce physicians’ workload positively influence physicians’ willingness to use computerized CPGs.

The dependent variable of this study is physicians’ intention to use computerized CPGs, and is operationalized as the strength of an individual’s willingness to take a certain action [62, 71]. The higher the strength of a behavioral intention is, the higher the likelihood that the action will be taken. Behavioral intention measures the degree of willingness to perform a certain action. In this study, physicians’ intention to use computerized CPGs is defined as physicians’ internal orientation toward and motivation to apply computerized CPGs. As discussed, we proposed eight hypotheses that can be divided into three categories.

1. Tool (technology) factors have a significant impact on physicians’ intentions to use computerized CPGs.
  1. The complexity of computerized CPGs affects physicians’ intention to use computerized CPGs.
  2. The perceived usefulness of computerized CPGs affects physicians’ intention to use computerized CPGs.
  3. The compatibility of computerized CPGs affects physicians’ intention to use computerized CPGs.
  4. The perceived ease of use of computerized CPGs affects physicians’ intention to use computerized CPGs.
2. Subject (human) factors have a significant impact on physicians’ intention to use computerized CPGs.
  H2a: Physicians’ attitude affects their intention to use computerized CPGs.
  H2b: The task uncertainty faced by physicians affects their intention to use computerized CPGs.
3. Environment (organization) factors have a significant impact on physicians’ intention to use computerized CPGs.
  H3a: Social influence affects physicians’ intention to use computerized CPGs.
  H3b: Organizational support affects physicians’ intention to use computerized CPGs.

### Instrument and subjects

The initial research framework and corresponding questionnaires were developed according to a literature review and revised by a panel consisting of four experts (two directors of an EBM center, one attending physician practicing clinical medicine, and one professor with a master’s degree in medical information) to increase content validity. Expert validity was measured using a content validity index (CVI) of 0.8 as the criterion for item selection [77, 78]. All CVI values of measurement items, except for one item, exceeded 0.8, and the overall CVI was 0.96, indicating excellent expert validity. In addition, the experts provided necessary semantic revisions to ensure that the retained items were appropriate and consistent. The revised questionnaire was then pretested by five physicians who were not employees of the investigated hospitals to correct semantic misunderstandings before distribution to the respondents. To address potential ethical concerns, our study protocol and informed consent forms were reviewed and approved by the Institutional Review Board (IRB) of the Chi-Mei Medical Center in Taiwan before the surveys were distributed and collected. After receiving approval from the IRB of the target hospital (IRB-10206-006), research was conducted from 13 June 2013 to 14 June 2014. Study participants were voluntary and verbal consent (waiver documentation of consent). Responses were anonymous and untraceable to individual physician.

We collected data from physicians of the three investigated hospitals (one medical center and two regional hospitals) that have established EBM centers. The reason for the selection of the case hospitals is that EBM centers are the major units promoting clinical guidelines and providing clinical guidelines related to training and education courses for physicians in Taiwanese hospitals. The EBM is a candidate course on health care quality in physicians’ continuing education, and EBM has been a fundamental training course for postgraduate year 1 (PGY1) resident physicians since 2003. The PGY1 training courses are mandatory and require resident physicians to complete 3–6 h of training in using EBM databases and at least eight clinical case analysis reports on EBM topics in 2011 [5]. Furthermore, the investigated hospitals are pilot adopters of computerized CPGs in Taiwan, and they have implemented computerized CPGs by incorporating them into existing HIS and EMR systems to provide timely clinical decision support since 2011. Because this study investigated critical factors influencing physicians’ intention to use computerized CPGs rather than the actual use of computerized CPGs, all physicians of the investigated hospitals were potential respondents.

This study employed a survey methodology that involved using a 41-item structured questionnaire composed of the following two major parts: (1) a part for recording the respondents’ demographic data, and (2) a part evaluating factors influencing physicians’ intention to use computerized CPGs. The questionnaire items were rated using a 5-point Likert scale, with scores ranging from 1 (*strongly disagree*) to 5 (*strongly agree*).

The tool construct (technology characteristics) addressed complexity, perceived usefulness, compatibility, and perceived ease of use. Complexity was measured using four items adapted from previous studies [74, 76, 79]. Perceived usefulness was measured using five items adapted from previous studies [80–82]. Compatibility was measured using four items adapted from Rogers [63] and Teng et al. [65]. Perceived ease of use was measured using three items adapted from prior studies [58, 65, 67]. The subject construct (human characteristics) comprised attitude and task uncertainty. Attitude was measured using six items adapted from previous studies [21, 74, 83]. Task uncertainty was measured using three items adapted from previous studies [74, 84]. The environment construct (organization characteristics) comprised social influence and organizational support. Social influence was measured using four items adapted from Venkatesh et al. [62, 75]. Organizational support was measured using three items adapted from Cabana et al. [21] and Simpson et al. [76]. Physicians’ intention to use computerized CPGs was measured using two items adapted from Bhattacherjee and Hikmet [85]. Detailed descriptions of the questionnaire are shown in the Appendix.

### Data analysis

Structural equation modeling is an extension of several multivariate techniques (most notably multiple regression and factor analysis) that has been widely used in many research areas such as education, psychology, sociology, management, and health. ([86], p. 578). Hair et al. [86] summarized the reasons for the wide use of this technique twofold: (1) It provides a straightforward method for addressing multiple relationships simultaneously while enabling statistical efficiency, and (2) it assesses relationships comprehensively and provides a transition from exploratory to confirmatory analysis. We conducted confirmatory analysis to investigate the factors influencing physicians’ intention to use computerized CPGs. A structural equation modeling technique was used as the tool for further data analysis. The reliability and validity of the measurement model were assessed by conducting CFA with LISREL 8.5, and the maximum likelihood method was applied to estimate the parameters of the research model. A structural model was used to examine the causal model of the investigated model.

## Results

### Demographic data

The survey was distributed to 505 physicians of the three investigated hospitals, and 238 valid responses were returned, representing a response rate of 47.1 %. Voluntary participation by physicians and top management support might explain the relatively high response rate compared with the average rate (10–20 %) for physicians in Taiwan [87]. Most of the respondents were male (87.8 %), and all of them had a bachelor (73.9 %) or master’s (or higher) degree (26.1 %). Those aged between 31 and 50 years accounted for the highest proportion of the respondents (70.2 %). The major service departments of the respondents were other specialties (44.5 %), internal medicine (26.5 %), surgery (20.2 %), and obstetrics and pediatrics (8.6 %). Almost half of the respondents (52.9 %) had 10 or more years of experience in clinical practice at their current hospital. Most of the respondents (92.9 %) had previously used EBM databases, and 68.5 % had 3 years or more of experience in using CPGs. Approximately 62.6 % of the respondents had previously used CPGs, and 44.1 % had more than 1 year of experience of computerized CPG use. Therefore, most of the respondents were experienced users of computerized CPGs and EBM databases, and all of them had considerable experience in clinical practice. The representativeness of the respondents was thus demonstrated. The demographic data of the respondents are shown in Table 2.

**Table 2:** Participant demographic data

Measure	Category	No (#)	Percent (%)
Age	<30	16	6.7
	31–40	80	33.6
	41–50	87	36.6
	51–60	48	20.2
	>60	7	2.9
Gender	Male	209	87.8
	Female	29	12.2
Education level	Bachelor	176	73.9
	Master (or higher)	62	26.1
Department	Internal medicine	63	26.5
	Surgery	48	20.2
	Obstetrics and pediatrics	21	8.8
	Others	106	44.5
Years of experience in clinical practice	<1	2	0.8
	1–5	43	18.1
	5–10	67	28.2
	>10	126	52.9
Years of experience in using EBM	None	17	7.1
	<1	23	9.7
	1-3	35	14.7
	>3	163	68.5
Years of experience in using CCPG	None	89	37.4
	<1	44	18.5
	1–3	58	24.4
	>3	47	19.7

### Key factors contributing to physicians’ intention to CPGs use

#### Measurement model

The model comprised nine variables (complexity, perceived usefulness, compatibility, perceived ease of use, attitude, task uncertainty, social influence, organizational support, and intention to use computerized CPGs) with a total of 34 items. Seven model-fit measures were used to evaluate the overall goodness fit of the model and collected data as follows: the ratio of the chi-square (*χ* ^2^) to degree-of-freedom (df); goodness-of-fit index (GFI); normalized fit index (NFI); nonnormalized fit index (NNFI); incremental fit index (IFI); comparative fit index (CFI); and root mean square error of approximation (RMSEA). The results of goodness-of-fit statistics are summarized in Table 3. The ratio of the chi-square (*χ* ^2^ = 691.01) to degree-of-freedom (df = 263.00) was 2.63 for the measurement model. The value of GFI was 0.81, which is slightly lower than the recommended value but acceptable; the values of the NFI (0.90), NNFI (0.90), IFI (0.90), and CFI (0.90) were equal to the suggested 0.9 benchmark [86]. The RMSEA was 0.076, which is within the acceptable levels of 0.05–0.08 [86]. Therefore, most of the fit indices met the recommended values, indicating a reasonable fit between the data and the proposed measurement model.

**Table 3:** Model evaluation - overall fit measurement

Fit indices	Recommended value	Value
χ^2^	N/A	691.03
d.f	N/A	263
χ^2^/d.f	≤3.0	2.63
Goodness-of-fit index (GFI)	≧0.9	0.81
Normalized fit index (NFI)	≧0.9	0.90
Non-normalized fit index (NNFI)	≧0.9	0.90
Incremental fit index (IFI)	≧0.9	0.90
Comparative fit index (CFI)	≧0.9	0.90
Root mean square error of approximation (RMSEA)	≤0.08	0.076

The results of Cronbach’s α, composite reliability (CR), convergent validity, and discriminant validity are shown in Table 4. The values of Cronbach’s α for all constructs were higher than the 0.7 threshold and ranged from 0.702 to 0.927. The internal consistency of the measurement model by evaluating CR of the constructs ranged from 0.762 to 0.913, exceeding the recommended value of 0.70. Fornell and Larcker [88] suggested that the value of average variance extracted (AVE) exceed 0.5 and be greater than each square correlation, to indicate adequate convergent validity and discriminant validity. As shown in Table 4, the AVE of the measurements ranged from 0.580 to 0.832, indicating excellent convergent validity. This study also revealed that the square roots of all AVE values were greater than the off-diagonal elements presented in the corresponding rows and columns, suggesting excellent discriminant validity. Therefore, this study demonstrated adequate reliability, convergent validity, and discriminant validity.

**Table 4:** Results of reliability and validity of the research model

Variable	CMP	PU	COM	PE	PA	TU	SI	OS	ITU	AVE	CR	Cronbach’s α
CMP	**0.790**									0.621	0.868	0.853
PU	0.577	**0.762**								0.580	0.805	0.821
COM	0.689	0.614	**0.864**							0.747	0.923	0.894
PE	0.735	0.576	0.805	**0.829**						0.687	0.869	0.835
PA	0.208	0.392	0.269	0.342	**0.840**					0.706	0.878	0.702
TU	0.100	0.096	0.090	0.005	0.261	**0.835**				0.698	0.820	0.789
SI	0.206	0.450	0.486	0.424	0.115	0.077	**0.912**			0.832	0.908	0.891
OS	0.353	0.521	0.501	0.482	0.301	0.117	0.602	**0.893**		0.798	0.922	0.927
ITU	0.359	0.636	0.485	0.489	0.386	0.122	0.548	0.696	**0.868**	0.753	0.859	0.846

The bold numbers in the diagonal row are square roots of the AVE. Off-diagonal elements are the correlations among constructs

*Abbreviations*: *CMP* complexity, *PU* perceived usefulness, *COM* compatibility, *PE* perceived ease of use, *PA* physicians’ attitude, *TU* task uncertainty, *SI* social influence, *OS* organizational support, *ITU* intention to use computerized CPGs

#### Structure model

The path coefficient and R^2^ value were estimated primarily to test the structural model. The path coefficient represents the magnitude and direction of the relation vector between the variables and is used to test their significance; the R^2^ value refers to the extent to which the exogenous (independent) variables explain the variance of the endogenous (dependent) variables (in percentages), representing the predictive power of the model. Both the path coefficient and R^2^ value showed the fit between the structural model and empirical data.

As shown in Fig. 1, four hypotheses were supported significantly in this study. The results indicated that the perceived usefulness of computerized CPGs (γ = 0.219, *p <* 0.05) in tool characteristics, attitude toward using computerized CPGs (γ = 0.451, *p <* 0.001) in subject characteristics, and social influence (γ = 0.213, *p <* 0.05) and organizational support (γ = 0.285, *p <* 0.001) in environment characteristics were critical factors influencing physicians’ intention to use computerized CPGs, supporting H1b, H2a, H3a, and H3b. These factors accounted for 68.6 % of the total explained variance in physicians’ intention to use computerized CPGs. However, the data showed that the hypotheses regarding complexity (H1a), compatibility (H1c), perceived ease of use (H1d) in tool factors, and task uncertainty (H2b) in subject factors were not significantly supported.

## Discussion

The results show that the perceived usefulness of computerized CPGs, attitude toward using computerized CPGs, social influence, and organizational support are critical factors influencing physicians’ intention to use computerized CPGs. Consistent with Wu et al. [56], our study revealed that attitude is the most critical factor affecting physicians’ intention to use computerized CPGs. Cabana et al. [21] reported that identification with CPGs is the primary cause that hinders the promotion of computerized CPGs. Previous studies have suggested that a lack of knowledge, familiarity, and recognition indicates a negative physician attitude toward computerized CPGs [21, 74]. To improve physicians’ attitude toward computerized CPGs, hospital managers should expend substantial effort in computerized CPG training and education.

Perceived usefulness refers to users’ subjective beliefs about the benefits of using HIT to achieve job goals in medical practice [64, 65]. Stoddard et al. [89] found that when clinical guidelines are flexible and can be adjusted according to the situation to improve the quality of medical decisions, physicians are more willing to use the guidelines. Consistent with previous studies investigating health care professionals’ acceptance of HIT [58–61, 65, 67], our study demonstrated that perceived usefulness is a significant factor influencing physicians’ intention to use computerized CPGs. This finding implies that when physicians consider that actual benefits can be obtained through computerized CPG use, they have a higher intention to use such systems. We suggest that hospital managers establish a dedicated EBM team for promoting and educating physicians about the benefits of computerized CPGs to improve the quality of medical decisions, job performance, and cost effectiveness.

Our study showed that support from colleagues and supervisors positively affects physicians’ intentions to use computerized CPGs; this is consistent with the findings of Davis and Taylor-Vaisey [79] and Kortteisto et al. [41]. Thus, support from colleagues and supervisors is encouraged for facilitating the widespread use of computerized CPGs in a health care institute. Organizational support refers to resources (time, money, and human resources) provided by health care institutions to physicians to alleviate stress during computerized CPG use. Cabana et al. [21] and Simpson et al. [76] have reported that deficiency in organizational resources (e.g., personnel and time) increases workloads and personal work stress, leading to poor results of clinical guideline implementation. This finding is consistent with our results. Therefore, hospitals managers can alleviate physicians’ stress during computerized CPG use by providing sufficient personnel, time, and equipment, thereby increasing physicians’ intention to use computerized CPGs.

Although several critical factors influencing physicians’ intention to use computerized CPGs were identified, complexity, compatibility, perceived ease of use, and task uncertainty had no significant effect on physicians’ intention to CPG use. Simpson et al. found that higher patient disease complexity may lower the rate of computerized CPG adoption. A computerized CPG that accounts for various clinical conditions simultaneously has not been developed. In a follow-up interview with physicians, the experts expressed that physicians consider various conditions when making clinical decisions and cannot completely accept every suggestion provided by computerized CPGs. Thus, computerized CPGs may provide insufficient support when physicians are faced with complex decision-making situations. In addition, when clinical guidelines become increasingly comprehensive and interdisciplinary, their complexity inevitably increases.

Our study derived a result inconsistent with those of previous studies, in that compatibility was a critical factor affecting users’ willingness to adopt innovative technology [68, 69]. In 2009, the Ministry of Health and Welfare added EBM to the evaluation criteria for the Evaluation Standards and Measurement Criteria for Teaching Hospitals. EBM practices and the writing of medical records were included in the training of teachers, medical interns, and resident physicians. EBM learning and application were also incorporated into training for general medical skills. Thus, to manage and improve the quality of medical care, EBM standards should be used to determine whether the quality meets the required standards. In addition, regular reviews should be conducted. Therefore, knowledge regarding EBM is now included in the basic training for medical interns and general medical education; hence, the number of physicians who have read and understood the clinical guidelines has gradually increased. Moreover, several developed clinical guidelines have been combined with EMR and HIS to assist physicians in clinical decision-making processes. This can provide a possible explanation for why compatibility does not significantly affect physicians’ intention to use computerized CPGs.

Although perceived ease of use was found to be a critical factor influencing the user acceptance of systems among various health care professionals [58, 65, 67], perceived ease of use had no significant influence in our study. Moreover, Bhattacherjee and Hikmet ([85], p. 734) found that perceived ease of use nonsignificantly influenced physicians’ intention to use HIT. They argued that the effect of perceived ease of use can be mediated by other factors such as perceived usefulness [55] and perceived technology control [90]. Therefore, perceived ease of use may not be influential at the postimplementation stage of CPG use.

Task uncertainty refers to the frequency at which physicians encounter uncertainties and disorganization-related problems. Raymond and Bergeron [91] indicated that when encountering task uncertainty, system users exhibited increased satisfaction toward personalized decision-making systems. Although much attention has been paid to clinical guidelines in assisting physicians’ decision-making processes, task uncertainty was not a critical factor significantly influencing physician’ intention to use computerized CPGs. In practice, this finding may be attributed to three reasons: (1) When providing clinical care, physicians frequently encounter sudden, unexpected problems; (2) not all hospital departments have guidelines that can be followed to manage such problems; and (3) clinical care work can involve unprecedented diseases or complications, and treating patients generally requires that physicians from other disciplines participate and share their expertise. Although comprehensive and fully integrated cross-department clinical guidelines facilitate addressing aforementioned problems, few effective complex cross-departmental clinical guidelines have been developed. In addition, ANOVA analysis was conducted to investigate the effects of age and computerized CPG experience on physicians’ intention to use computerized CPGs. The results demonstrated that age and computerized CPG experience were significant demographic factors influencing physicians’ intention to use computerized CPGs. Although age and user experience were found to be significant to user intention in this study, future research can focus on exploring the moderate effect between the investigated factors and user intention.

The findings of this study are subject to five major limitations. First, we examined three hospitals with EBM centers belonging to one medical group in Southern Taiwan, to investigate organizational concerns (culture differences and the establishment of EBM centers), thus potentially restricting the generalization of the findings to other medical institutions. Second, data were collected from experienced users of EBM databases and computerized CPGs. The results from the respondents were based on users’ perceptions, experiences, and understanding. Thus, the data collected may not be adequately objective. Third, we analyzed only cross-sectional data collected by physicians during one period. The data should be carefully interpreted regarding the effect of time. Fourth, multimorbidity is one of the major causes inhibiting the development of CPGs. Because the 17 developed CPGs in Taiwan focus on a single disease rather than multiple conditions of patients, the findings might be not adequately applied in computerized CPGs for addressing multimorbidity problems. Finally, computerized CPG use in the three investigated hospitals is voluntary; thus, tracing individual physicians is difficult. This study investigated physicians’ intention to use computerized CPGs rather than actual use, because we could not directly identify the actual users of computerized CPGs. Two conditions may have caused this problem: (1) Some medical specialists (departments) do not have access to developed computerized CPGs, and (2) some physicians are computerized CPG users but do not consider themselves actual computerized CPG users because they may violate or neglect the suggestions from computerized CPGs. Future studies should pay particular attention to the mentioned problems.

## Conclusions

### Crucial findings

This study investigated critical factors influencing physicians’ intention to use computerized CPGs through an integrated model derived from activity theory and the TAM. This model comprising tool (technology) characteristics (complexity, usefulness, compatibility, and ease of use), subject (human) characteristics (attitude and task uncertainty), and environment (organization) characteristics (social influence and organizational support) was proposed and validated. The results showed that attitudes toward using computerized CPGs (γ = 0.451, *p* < 0.001), organizational support (γ = 0.285, *p* < 0.001), perceived usefulness of computerized CPGs (γ = 0.219, *p* < 0.05), and social influence (γ = 0.213, *p* < 0.05) were critical factors influencing physicians’ intention to use computerized CPGs, and these factors can explain 68.6 % of the variance in intention to use computerized CPGs. Managers should pay considerable attention to these identified factors and provide adequate resources and incentives to facilitate the promotion and use of computerized CPGs. The appropriate use of computerized CPGs will enable realizing clinical benefits, particularly by improving the quality of care and facilitating clinical processes. Computerized CPGs, which incorporate the advantages of both IT and CPGs, are considered effective means of facilitating the use of CPGs and improving the quality of care at affordable costs [23, 31]. This study is among the few computerized CPG studies that have emphasized management concerns. The findings of this study are useful in formulating appropriate strategies for increasing user participation at various system implementation stages (computerized CPG design, implementation, and postimplementation).

### Theoretical and practical implications

From a theoretical perspective, our study integrated the concepts of activity theory and the TAM to address the research gap created by previous IS/IT studies focusing excessively on technical-economic perspectives; thus, we propose a more comprehensive model for effectively explaining the phenomena relating to changes in health care IT use behavior, as suggested by Kuutti [44]. Evaluation based on activity theory perspective can provide insights into the potential reasons for and factors influencing computerized CPG use according to the subject, environment, and tool dimensions, and these factors are critical for realizing computerized CPG benefits (objects). Thus, activity theory can be applied to elucidate the interaction among individuals, tools, and the environment (at the organizational, societal, and other levels). This study formulated an integrative research model for investigating critical factors influencing HIT adoption according to activity theory and TAM results. The proposed research model can be extended and used to identify critical factors influencing the adoption of HIT and clinical decision support systems in the health care industry.

The increasing emphasis on EBM has transformed the concept of EBM from a mere topic in academic research to a method applied in clinical practice. Empirical evidence-based decision models can be used to assist decision makers in establishing policies based on priorities and the efficient use of limited resources; thus, these models are vital decision-making tools [82]. In practice, implementing computerized CPG influences both the medical care procedures and patient care habits of physicians; therefore, the identified key factors contribute to physicians’ willingness to use computerized CPGs. By understanding the critical factors, further analysis can be conducted and effective strategies can be formulated to facilitate the widespread use and promotion of computerized CPGs.

Previous studies have found that introducing computerized CPGs may change physicians’ clinical behaviors and result in resistance to computerized CPGs use [20, 21]. Our study showed that physicians’ attitude toward computerized CPGs is the most crucial factor affecting their intention to use computerized CPGs. In addition, our study demonstrated that perceived usefulness is a significant factor influencing physicians’ intention to use computerized CPGs. This finding indicates that when physicians consider that actual benefits are obtained through computerized CPG use, they have a higher intention to use the system. Effectively educating physicians and advocating the computerized CPG use could reinforce the usefulness of clinical guidelines, improve medical decision adequacy, enable clinical cost effectiveness, and ultimately improve physicians’ intention to use computerized CPGs.

Our study confirmed that social influence and organizational support are critical for physicians’ intention to use computerized CPGs. Supervisor support and colleague recommendations regarding computerized CPG use are helpful for enhancing physicians’ intention to use CPGs. The results demonstrated that providing physicians with sufficient resources (time, money, and human resources) facilitates alleviating stress during computerized CPGs use. Therefore, hospital managers can alleviate physicians’ stress from using computerized CPGs by providing sufficient personnel, time, and equipment, thereby increasing physicians’ intention to use computerized CPGs.

## Appendix

**Table 5:** Questionnaire

Item no.	Item description
1. CMP1	The clinical guidelines provided by computerized CPGs is easy to understand.
2. CMP2	The clinical guidelines provided by computerized CPGs is easy to be tried.
3. CMP3	The use of computerized CPGs does not limited to specific tools and supports.
4. CMP4	The use of computerized CPGs does not need extra assistants from specific IT personnel.
5. PU1	The use of computerized CPGs can improve patient care quality.
6. PU2	The use of computerized CPGs can improve work efficiency.
7. PU3	The computerized CPGs is useful for training and education activities.
8. PU4	The computerized CPGs is helpful in providing adequate care quality within affordable costs.
9. PU5	The computerized CPGs can provide sufficient medical evidence for protecting physicians when lawsuits occurred.
10. COM1	Using computerized CPGs is compatible with existing electronic medical record systems.
11. COM2	The computerized CPGs is compatible with existing computer hardware and devices.
12. COM3	The computerized CPGs is compatible with my job characteristic.
13. COM4	The computerized CPGs is compatible with my working processes.
14. PE1	Learning to use computerized CPGs is easy for me.
15. PE2	The computerized CPGs provides adequate instruments in assisting its use.
16. PE3	It is easy to use computerized CPGs in obtaining required patient information.
17. ATT1	Using computerized CPGs will inhibit the innovation in clinical diagnosis and treatment.
18. ATT2	Using computerized CPGs will challenge physicians’ autonomy.
19. ATT3	The computerized CPGs is inflexible and hard to be applied to deal with the problems of individual patients.
20. ATT4	The computerized CPGs can help to deal with complex decision-making problems.
21. ATT5	The computerized CPGs can improve the quality of patient care.
22. ATT6	The computerized CPGs can facilitate the relationships between physicians and patients.
23. TU1	When executing my clinical task, the level of variety in regard to situations, intervening individuals and activities is high.
24. TU2	There are often ad-hoc problems encountered in my job and less guidelines can be used to deal with those problems.
25. TU3	The execution of my task usually deals with new problems.
26. SI1	Top managers support the use of computerized CPGs.
27. SI2	Top managers encourage to use computerized CPGs in our job processes.
28. SI3	I will use the computerized CPGs when my colleagues and peers think I should use computerized CPGs.
29. SI4	My colleagues and peers recognized the use of computerized CPGs is a right decision for me.
30. OS1	It can reduce my workload when the organization provides sufficient human resources.
31. OS2	I will use computerized CPGs to minimize my work pressures when the organization provides sufficient time of patient care.
32. OS3	I will use the computerized CPGs in improving clinical diagnosis and treatment activities when the organization provides adequate medical equipment and devices.
33. ITU1	I intend to use computerized CPGs when clinical decision making is required.
34. ITU2	The computerized CPGs will be my major tools in assisting my clinical diagnosis and treatment activities.
